# Development of a voltammetric analytical method for the quantification of mercury in urine

**DOI:** 10.17843/rpmesp.2026.431.14912

**Published:** 2026-03-30

**Authors:** Manuel Chavez-Ruiz, Ruddy Mesa-Landeo, Lenin Rueda-Torres, Betsabe Ruffner-Camargo, Sabir Khan

**Affiliations:** 1 National Center for Occupational Health and Environmental Protection for Health (CENSOPAS), Instituto Nacional de Salud, Lima, Peru.; 2 Universidade Federal Rural do Semi-Arido, Engineering Center, Rio Grande do Norte, Brazil.

**Keywords:** Mercury, Electrochemistry, Urine, Exposure to Chemical Compounds

## Abstract

Exposure to mercury represents a critical challenge for public health, necessitating precise and portable analytical methods for biomonitoring. This experimental analytical study develops an analytical method and evaluates the performance of square wave anodic stripping voltammetry (SWASV) for the quantification of mercury in urine. For this purpose, the operating conditions of the SWASV system were optimized through tests with standard mercury solutions. Commercial urine (BIO-RAD®) pretreated with nitric acid was used. Linearity, precision, trueness, limit of detection (LOD), and limit of quantification (LOQ) were evaluated. The results showed high precision, with a relative standard deviation (%RSD) of 6.21. Furthermore, the trueness analysis revealed no significant differences between SWASV and the cold vapor atomic absorption (CVAA) method, p = 0.2601. The LOD and LOQ were 0.68 and 2.2 µg L^-1^, respectively. These findings suggest that SWASV is a precise method comparable to CVAA for the detection of mercury in urine under controlled laboratory conditions. Additional studies are required to evaluate its applicability in real population biomonitoring scenarios.

## INTRODUCTION

Mercury is one of the chemical substances of greatest concern for public health ^1^. This environmental contaminant is ubiquitous, neurotoxic, and bioaccumulative, and its presence in ecosystems has been intensified by anthropogenic activities such as mining ^2^. It is estimated that between 14 and 19 million artisanal miners worldwide are directly exposed to mercury ^3^, while in Peru, evidence of genotoxic damage has been found in artisanal gold mining workers where mercury is used ^4^.

Mercury biomonitoring in urine is a fundamental tool for the surveillance and early identification of exposed individuals ^5^^,^^6^. This indicator is widely used in biomonitoring workers exposed to inorganic mercury; likewise, it could be applied in the monitoring of communities with high fish consumption, in which case other indicators such as hair or blood should be considered ^7^.

Mercury exposure is especially critical during vulnerable stages of human development, such as the prenatal period and childhood, given that neurological alterations have been documented even at urinary mercury concentrations lower than reference values ^7^. The World Health Organization (WHO) establishes a lower threshold of 7 μg Hg L⁻¹ as a "safety" level, while concentrations exceeding 25 μg Hg L⁻¹ require intervention ^8^.

Reference analytical methods for quantifying metals require high costs in equipment, maintenance, supplies, and specialized personnel ^9^, often making them unfeasible for resource-limited environments. Electrochemistry has emerged as a promising alternative to overcome these limitations. In particular, square wave anodic stripping voltammetry (SWASV) stands out among electroanalytical methods for the detection of heavy metals ^10^.

A voltammetric system is composed of an electrochemical detection instrument with three electrodes: a working electrode (WE), where oxidation and reduction reactions (Faraday reactions) of the species in the electrolytic solution occur; a reference electrode (RE), which maintains a constant and stable potential; and a counter electrode (CE), which closes the electrical circuit and allows current flow ^11^. The process consists of applying an electrical potential to the WE that induces redox reactions at its interface. The resulting current, generated by the transfer of electrons, is proportionally related to the concentration of the analyte in solution ^12^.

Additionally, SWASV incorporates a process of preconcentration of metal ions on the surface of the WE, followed by the application of symmetric potential pulses in the form of a square wave to excite the electrode ^13^. During this process, the metal previously deposited on the WE undergoes oxidation and is released back into the solution in a phenomenon known as anodic stripping. This generates a current peak whose intensity provides this technique with high sensitivity for the detection of trace metals ^14^.

Recent literature highlights the superiority of SWASV in terms of sensitivity and precision compared to conventional techniques ^15^^,^^16^. These factors must be evaluated and optimized to maximize the current signal and improve the precision of the analysis. On the other hand, the complexity of the biological matrix represents an additional challenge. Urine contains multiple interfering substances that can affect the electrochemical response, compromising the accuracy and reliability of measurements ^17^. In this context, the present study aims to develop and propose a SWASV analytical method for the detection of mercury in urine samples.

**KEY MESSAGES**

Motivation for conducting the study. Mercury exposure is a public health problem in Peru. It is necessary to have accessible analytical methods for mercury biomonitoring in humans.

Main findings. The developed voltammetric method showed adequate analytical performance for the quantification of mercury in urine samples, with high precision, good accuracy compared to a reference method, and detection limits compatible with biomonitoring applications using high-precision laboratory equipment.

Public health implications. Voltammetry could constitute a complementary analytical tool for mercury biomonitoring in urine, contributing to the strengthening of human exposure surveillance in occupational and environmental contexts.

## THE STUDY

An experimental analytical study was developed under controlled laboratory conditions, aimed at evaluating the analytical performance parameters of the SWASV method, including linearity, precision, trueness, and limits of detection and quantification.

### Preparation of solutions and samples

For the calibration and optimization of electrochemical parameters, a standard solution of mercury (II) (10 μg L⁻¹) was prepared using mercury (II) chloride (MERCK); a 0.0125 mol L⁻¹ hydrochloric acid solution (JT BAKER) was used as the supporting electrolyte.

As a urine sample, commercial lyophilized human urine Lyphochek BIO-RAD (United States; Level 1; Lot 69221) was used, which contains known concentrations of mercury and other metals indicated in its certificate of analysis. The urine sample, previously reconstituted with water, is prepared according to the following procedure: 5 mL of urine were mixed with 5 mL of 65% concentrated nitric acid (JT BAKER) and 3 mL of 10 mmol L⁻¹ potassium permanganate (MERCK). The mixture was subjected to thermal pretreatment at 70 °C for 30 minutes to ensure complete oxidation of organic matter. Subsequently, the solution was cooled to room temperature and diluted to a final volume of 15 mL with deionized water, and finally transferred to the electrochemical cell for analysis by SWASV.

### Voltammetric system

**Figure 1:**
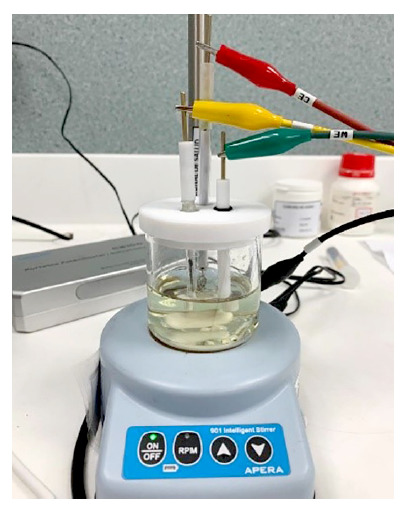
Voltammetric system developed with three electrodes (WE: green, CE: red, RE: yellow), on a magnetic stirrer and connected to a portable potentiostat (Corrtest).

The developed system is composed of an electrochemical cell with a 20 mL capacity for the electrolyte and includes three electrodes: a 4 mm diameter gold WE, a platinum counter electrode, and an Ag/AgCl RE immersed in KCl. In voltammetry, the gold electrode is preferred for quantifying mercury because Hg has a high affinity for Au and forms an Au-Hg amalgam, allowing for efficient preconcentration of the analyte on the electrode surface; this generates more intense, well-defined, and reproducible stripping peaks. These electrodes were connected to a potentiostat (Corrtest, China; model CS2350M), responsible for regulating the potential difference between the WE and the RE. The cell was placed on a magnetic stirrer (APERA Instruments, China; model 901) to ensure adequate homogenization of the analyte in the solution (Figure 1).

Briefly, the voltammetric analysis of mercury is carried out in two stages. In the first, a reduction potential is applied to electrodeposit the dissolved mercury (II) onto the surface of the WE (reaction 1). In the second stage, anodic stripping of the mercury is performed by applying a square wave potential scan in the direction of oxidation (reaction 2). As the mercury oxidizes, electrons are released, generating current intensity peaks (amperes), which are recorded and analyzed as a measure proportional to the mercury concentration.

Hg^+2^ + 2e^-^ ⇄ Hg^0^ (reaction 1)

Hg^0^ + 2Cl^-^ ⇄ ↓ HgCl_2_ + 2e^-^ (reaction 2)

The actual electrochemical measurements are performed after adjusting their operating parameters. These include deposition potential and time, step potential, amplitude modulation, and frequency. These parameters were defined with the standard mercury (II) solution (10 μg L⁻¹) in hydrochloric acid (0.0125 mol L⁻¹) as the supporting electrolyte.

Between each measurement, the electrochemical cell was cleaned with a nitric acid solution and rinsed with deionized water. All measurements were performed at room temperature (24 ± 2 °C).

### Optimization and preliminary evaluation of analytical performance

For the optimization of the electrochemical system, the mercury (II) standard solution was used. The choice of each parameter of the electrochemical method was taken as the value with the best current intensity (Ip) response recorded by the system and expressed in units of amperes. Different values of deposition potential, deposition time, amplitude, frequency, and step potential were tested. Three replicates were applied for all tests.

The analytical performance evaluated linearity, precision, trueness, LOD, and LOQ; for this, the recommendations of the Eurachem guide, 2016 version, were followed ^18^. To evaluate the precision of the method, repeatability was estimated by performing replicate analysis (n = 10) of the BIO-RAD urine control material under identical experimental conditions; the relative standard deviation (%RSD) was calculated. The trueness of the method was determined by comparing the results of four samples analyzed using the SWASV method with those obtained through the reference method, CVAA (cold vapor atomic absorption). To statistically compare the difference between both methods, a paired t-test was performed, after verification of data normality. A significance level of p < 0.05 was considered.

For the evaluation of linearity, a mercury (II) concentration versus response curve was constructed with five points and three replicates for each of the concentrations (2.5, 5, 10, 15, and 20 µg/L) in an aqueous HCl solution (c= 0.0125 mol L⁻¹). The LOD and LOQ were analyzed with seven urine samples spiked with mercury (II) at a concentration of 2.5 µg L⁻¹; equations 1 and 2 were applied respectively.

LOD = X + 3SD (Equation 1)

LOQ = X + 10SD (Equation 2)

## FINDINGS

### Electrochemical optimization parameters

**Figure 2:**
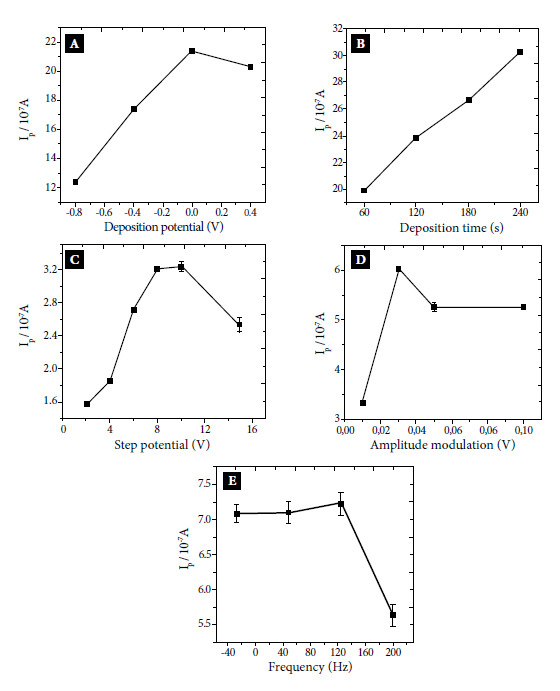
Optimization of the operating parameters of the VRAO: (A) deposition potential, (B) deposition time, (C) step potential, (D) modulation amplitude, and (E) frequency, in the voltammetric responses (stripping peak current) for mercury (II) in a 0.0125 mol L⁻¹ HCl solution. V: volts.

Figure 2 presents the results of the optimization of the SWASV system parameters. The optimal potential for mercury (II) deposition is observed in Figure 2A, where the current intensity peak is recorded at a potential of 0 volts (V), indicating the optimal signal for this parameter. The deposition time shows a positive linear relationship with current intensity (Figure 2B); however, excessively long times can saturate the WE with mercury, hindering its desorption during the oxidation stage. Therefore, an intermediate deposition time of 120 s was selected.

The step potential (Figure 2C) influences the scanning speed of mercury and significantly improves the sensitivity of the SWASV; an optimal value of 0.008 V was selected, as it provided the best analytical response for mercury (II) detection. The amplitude directly affects the height and width of the electrical intensity peaks; as shown in Figure 2D, values higher than 0.03 V in the modulation amplitude generated shorter peaks for mercury (II). Regarding frequency (Figure 2E), the tests indicated that its increase did not significantly affect the peak current intensity up to 150 Hz; however, a notable decrease was recorded at 200 Hz. Therefore, a frequency of 150 Hz was chosen.

**Figure 3:**
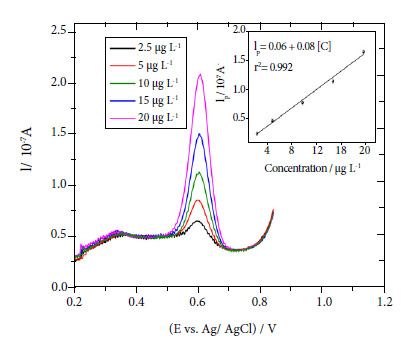
Response of the VRAO system with optimized parameters: deposition potential of 0.0 V, deposition time of 120 s, pulse amplitude of 0.03 V, step potential of 0.008 V, and frequency of 150 Hz. Calibration curve for the determination of mercury (II) in a concentration range of 2.5 to 20 µg L⁻¹.

Figure 3 shows the response of the SWASV system in current intensity (Ip) with the optimal parameters adjusted. The Ip increases linearly with the increase in mercury (II) concentration in a range of 2.5 to 20 µg L⁻¹ of mercury (II) (n = 5) with a correlation coefficient (R²) of 0.992 and a calibration curve equation: Ip (µA) = 0.8 C + 0.6. The standard deviation of seven urine samples spiked with mercury (II) (c = 2.5 µg L⁻¹) was 0.28. The LOD and LOQ were 0.68 and 2.2 µg L⁻¹, respectively.

The repeatability result for the SWASV method was 6.21 for %RSD, significantly lower than the theoretical threshold of 21.38 calculated using the Horwitz equation (2/3 %RSD Horwitz).

The results obtained by SWASV and CVAA are summarized in Table 1. The p-value obtained was 0.260, indicating that there is no significant difference between the mercury concentrations between both methods.

**Table 1:** Determination of mercury in BIO-RAD urine control samples (Level 1; Lot 69221) by SWASV and CVAA methods.

Sample	SWASV (μg L-1)	(CVAA μg L-1)	P-value
1	27.0	28.2	
2	30.5	29.1	
3	29.1	31.7	
4	25.7	31.2	
Mean (SD)	28.1 (2.1)	30.1 (1.7)	0.260^a^

a Paired sample Student's t-test

SWASV: square wave anodic stripping voltammetry, CVAA: cold vapor atomic absorption, SD: standard deviation.

## DISCUSSION

SWASV performance parameters were evaluated, focusing on linearity, precision, trueness, and limits of detection and quantification to ensure the reliability of the method. The results obtained from the urine control samples confirmed the performance and quality of the method under laboratory conditions for mercury quantification. The analytical method demonstrated satisfactory precision, complying with the criteria established by the Eurachem Guide (%RSD_Horwitz < 45%) ^18^. Similarly, intermediate precision values, expressed as a coefficient of variation, were less than 5%, which can be considered an acceptable value for a quantitative analytical method.

In contrast, RSD (%) values under repeatability conditions ranging from 0.3% to 3.6% have been reported using a carbon microfiber electrode modified with gold nanoparticles in water samples ^19^. However, a method has been developed for the digestion of mercury in urine samples using concentrated acid at high temperatures; this method demonstrated good repeatability, as confirmed by low standard deviations (<11%). Furthermore, its analysis verified that no loss of mercury occurred during acid digestion ^20^, similar to the current method, which reinforces the applicability of the proposed approach for mercury analysis in urine samples.

The observed repeatability of the SWASV method can be attributed to the electrochemical parameters, including deposition potential, deposition time, and frequency ^21^. These parameters were carefully selected to enhance the accumulation of mercury ions on the electrode surface, thereby improving the sensitivity and precision of the measurements. The use of a gold electrode, known for its stable electrochemical properties, further contributed to the robustness of the method ^22^. The agreement of results between SWASV and CVAA suggests that the voltammetric method is capable of achieving precision comparable to the spectroscopic technique, despite the inherent challenges associated with electrochemical measurements in complex biological matrices.

As limitations, it must be recognized that the present study uses commercial lyophilized urine controls; likewise, the useful life of the electrode and the drift of the applied system have not been determined. Linearity only covers the response in aqueous solution; the matrix effect has not been studied. Future studies on population samples will be needed to evaluate the applicability of the SWASV method in a wider range of conditions and matrices to establish its utility in diverse analytical contexts.

In conclusion, this study developed an analytical methodology applying SWASV, optimized the main analytical parameters, and performed a preliminary validation for the measurement of trace mercury in water and urine. The results demonstrate that SWASV is a method with adequate precision, comparable to the CVAA reference method and adequate LOD and LOQ values; added to the great advantage of being a portable method, it is granted high potential for its application as a tool in the evaluation of occupational exposure to mercury. Future research should focus on optimizing SWASV parameters to detect other trace metals and evaluate its applicability in various biological and environmental samples. Furthermore, the integration of SWASV with emerging technologies, such as microfluidic systems or nanomaterial-based sensors, could improve its sensitivity and selectivity, paving the way for innovative solutions in analytical chemistry.
