# Supplementary material for: Development of a voltammetric analytical method for the quantification of mercury in urine
Source: Rev Peru Med Exp Salud Publica. 2026 Mar 30;43(1):77–82. doi: 10.17843/rpmesp.2026.431.14912 (PMC13245983; doi:10.17843/rpmesp.2026.431.14912)
Supplement: Translation

# Translation: Desarrollo de un método analítico voltamperométrico para la cuantificación de mercurio en orina

**Authors:** Manuel Chavez-Ruiz, Ruddy Mesa-Landeo, Lenin Rueda-Torres, Betsabe Ruffner-Camargo, Sabir Khan

**Affiliations:** 1 Centro Nacional de Salud Ocupacional y Protección del Ambiente para la Salud (CENSOPAS), Instituto Nacional de Salud, Lima, Perú.; 2 Universidade Federal Rural do Semi-Arido, Centro de Engenharia, Rio Grande do Norte, Brasil.

**Keywords:** Mercurio, Electroquímica, Orina, Exposición a Compuestos Químicos

## Abstract

La exposición al mercurio representa un desafío crítico para la salud pública, por lo que es necesario contar con métodos analíticos precisos y portátiles para su biomonitoreo. Este estudio de tipo experimental analítico, desarrolla un método analítico y evalúa el desempeño de la voltamperometría de redisolución anódica de onda cuadrada (VRAO) para la cuantificación de mercurio en orina. Para ello se optimizaron las condiciones de funcionamiento del sistema VRAO mediante pruebas con soluciones estándar de mercurio. Se utilizó orina comercial (BIO-RAD®) pretratado con ácido nítrico. Se evaluó la linealidad, precisión, veracidad, el límite de detección (LDD) y de cuantificación (LDC). Los resultados mostraron una alta precisión, con una desviación estándar relativa (%DER) de 6,21. Además, el análisis de veracidad no reveló diferencias significativas entre VRAO y el método de absorción atómica de vapor frío (AAVF), p = 0,2601. El LDD y LDC resultaron 0,68 y 2,2 µg L^-1^, respectivamente. Estos hallazgos sugieren que VRAO es un método preciso y comparable con AAVF para la detección de mercurio en orina bajo condiciones controladas de laboratorio. Se requieren estudios adicionales para evaluar su aplicabilidad en escenarios reales de biomonitoreo poblacional.

## INTRODUCCIÓN

El mercurio es una de las sustancias químicas de mayor preocupación para la salud pública ^1^. Este contaminante ambiental es ubicuo, neurotóxico y bioacumulable, cuya presencia en los ecosistemas se ha intensificado por actividades antropogénicas, como la minería ^2^. Se estima que entre 14 y 19 millones de mineros artesanales en todo el mundo están expuestos directamente al mercurio ^3^, mientras que en el Perú se ha encontrado evidencia de daño genotóxico en trabajadores de minería artesanal de oro donde se emplea mercurio ^4^.

El biomonitoreo de mercurio en orina es una herramienta fundamental para la vigilancia e identificación temprana de expuestos ^5^^,^^6^. Este indicador es ampliamente usado en biomonitoreo en trabajadores expuestos a mercurio inorgánico, asimismo podría ser aplicado en monitoreos de comunidades con alto consumo de pescado, en este caso teniendo en cuenta otros indicadores como cabello o sangre ^7^.

La exposición a mercurio es especialmente crítica en etapas vulnerables del desarrollo humano, como el período prenatal y la infancia, dado que se han documentado alteraciones neurológicas incluso a concentraciones de mercurio urinario inferiores a los valores de referencia ^7^. La Organización Mundial de la Salud (OMS) establece un umbral inferior de 7 μg Hg L⁻¹ como un nivel de «seguridad», mientras que concentraciones superiores a 25 μg Hg L⁻¹ requieren intervención ^8^.

Los métodos analíticos referenciales que cuantifican metales, demandan altos costos en equipamiento, mantenimiento, insumos y personal especializado ^9^, resultando muchas veces inviables para entornos de recursos limitados. La electroquímica ha surgido como una alternativa prometedora para superar estas limitaciones. En particular, la voltamperometría de redisolución anódica de onda cuadrada (VRAO) destaca entre los métodos electroanalíticos para la detección de metales pesados ^10^.

Un sistema voltamperométrico está compuesto por un instrumento de detección electroquímico con tres electrodos: un electrodo de trabajo (ET), donde ocurren las reacciones de oxidación y reducción (reacciones de Faraday) de las especies en solución electrolítica; un electrodo de referencia (ER), que mantiene un potencial constante y estable; y un contraelectrodo (CE), que cierra el circuito eléctrico y permite el flujo de corriente ^11^. El proceso consiste en aplicar un potencial eléctrico al ET que induce reacciones redox en su interfase. La corriente resultante, generada por la transferencia de electrones, se relaciona proporcionalmente con la concentración del analito en solución ^12^.

Adicionalmente, la VRAO incorpora un proceso de preconcentración de iones metálicos en la superficie del ET, seguido de la aplicación de pulsos simétricos de potencial en forma de onda cuadrada para excitar el electrodo ^13^. Durante este proceso, el metal previamente depositado en el ET sufre oxidación y es liberado nuevamente a la solución en un fenómeno conocido como redisolución anódica. Esto genera un pico de corriente cuya intensidad confiere a esta técnica una alta sensibilidad para la detección de metales en trazas ^14^.

Literatura reciente destaca la superioridad de la VRAO en términos de sensibilidad y precisión en comparación con técnicas convencionales ^15^^,^^16^. Estos factores deben ser evaluados y optimizados para maximizar la señal de corriente y mejorar la precisión del análisis. Por otro lado, la complejidad de la matriz biológica representa un desafío adicional. La orina contiene múltiples sustancias interferentes que pueden afectar la respuesta electroquímica, comprometiendo la exactitud y confiabilidad de las mediciones ^17^. En este contexto, el presente estudio tiene como objetivo el desarrollo y la propuesta de un método analítico de VRAO para la detección de mercurio en muestras de orina.

**MENSAJE CLAVE**

Motivación para realizar el estudio. La exposición al mercurio es un problema de salud pública en Perú. Es necesario contar con métodos analíticos accesibles para el biomonitoreo de mercurio en seres humanos.

Principales hallazgos. El método voltamperométrico desarrollado mostró un adecuado desempeño analítico para la cuantificación de mercurio en muestras de orina, con alta precisión, buena exactitud frente a un método de referencia y límites de detección compatibles con aplicaciones de biomonitoreo con equipos de alta precisión de laboratorio.

Implicancias en salud pública. La voltamperometría podría constituir una herramienta analítica complementaria para el biomonitoreo de mercurio en orina, contribuyendo al fortalecimiento de la vigilancia de la exposición humana en contextos ocupacionales y ambientales.

## EL ESTUDIO

Se desarrolló un estudio experimental analítico, bajo condiciones controladas de laboratorio, orientado a la evaluación de los parámetros de desempeño analítico del método VRAO, incluyendo linealidad, precisión, veracidad, límites de detección y cuantificación.

### Preparación de soluciones y muestras

Para la calibración y optimización de los parámetros electroquímicos, se preparó una solución estándar de mercurio (II) (10 μg L⁻¹), para ello se usó cloruro de mercurio (II) (MERCK), como electrolito de soporte se usó una solución de ácido clorhídrico 0,0125 mol L⁻¹ (JT BAKER).

Como muestra de orina se empleó una orina humana liofilizada comercial Lyphochek BIO-RAD (Estados Unidos; Nivel 1; Lote 69221), la cual contiene concentraciones conocidas de mercurio y otros metales, indicadas en su certificado de análisis. La muestra de orina previamente reconstituida con agua, se prepara de acuerdo al siguiente procedimiento: Se mezclaron 5 mL de orina con 5 mL de ácido nítrico concentrado al 65% (JT BAKER) y 3 mL de permanganato de potasio 10 mmol L⁻¹ (MERCK). La mezcla se sometió a un pretratamiento térmico a 70 °C durante 30 minutos para asegurar la oxidación completa de la materia orgánica. Posteriormente, la solución se enfrió a temperatura ambiente y se diluyó hasta un volumen final de 15 mL con agua desionizada y finalmente se transfirió a la celda electroquímica para su análisis por VRAO.

### Sistema voltamperométrico

El sistema desarrollado se compone de una celda electroquímica de 20 mL de capacidad para el electrolito e incluye tres electrodos: un ET de oro de 4 mm de diámetro, un contraelectrodo de platino y un ER de Ag/AgCl sumergido en KCl. En voltamperometría, el electrodo de oro es preferido para cuantificar mercurio porque el Hg tiene alta afinidad por el Au y forma una amalgama Au-Hg, lo que permite una preconcentración eficiente del analito en la superficie del electrodo; esto genera picos de redisolución más intensos, bien definidos y reproducibles. Estos electrodos fueron conectados a un potenciostato (Corrtest, China; modelo CS2350M), encargado de regular la diferencia de potencial entre el ET y el ER. La celda se colocó sobre un agitador magnético (APERA Instruments, China; modelo 901) para garantizar una adecuada homogenización del analito en la solución (figura 1).

**Figura 1:** Sistema voltamperométrico desarrollado con tres electrodos (ET: verde, CE: rojo, ER: amarillo), sobre un agitador magnético y conectado a un potenciostato (Corrtest) portable.

Brevemente, el análisis voltamperométrico del mercurio se lleva a cabo en dos etapas. En la primera, se aplica un potencial de reducción para electrodepositar el mercurio (II) disuelto sobre la superficie del ET (reacción 1). En la segunda etapa, se realiza la redisolución anódica del mercurio mediante la aplicación de un barrido de potencial de onda cuadrada en dirección de oxidación (reacción 2). A medida que el mercurio se oxida, se liberan electrones, generando picos de intensidad de corriente (amperios), que son registrados y analizados como una medida proporcional a la concentración del mercurio.

Hg^+2^ + 2e^-^ ⇄ Hg^0^(reacción 1)

Hg^0^ + 2Cl^-^ ⇄ ↓ HgCl_2_ + 2e^-^(reacción 2)

Las mediciones electroquímicas propiamente dichas se ejecutan posterior al ajuste de sus parámetros de funcionamiento. Estos incluyen el potencial y tiempo de deposición, el potencial de paso, la modulación de amplitud y la frecuencia. Estos parámetros fueron definidos con la solución estándar de mercurio (II) (10 μg L⁻¹) en ácido clorhídrico (0,0125 mol L⁻¹) como electrolito soporte.

Entre cada medición, la celda electroquímica se limpia con una solución de ácido nítrico y se enjuagó con agua desionizada. Todas las mediciones se realizaron a temperatura ambiente (24 ± 2 °C).

### Optimización y evaluación preliminar del desempeño analítico

Para la optimización del sistema electroquímico se empleó la solución estándar de mercurio II, la elección de cada parámetro del método electroquímico se tomó como aquel valor con la mejor respuesta de la intensidad de corriente (I_p_) registrada por el sistema y expresada en unidades de amperios. Se probaron diferentes valores de potencial de deposición, tiempo de deposición, amplitud, frecuencia y potencial de paso. Para todos los ensayos se aplicó tres replicas.

El desempeño analítico evaluó, linealidad, precisión, veracidad, LDD y DLC; para ello se siguieron las recomendaciones de la guía Eurachem, versión 2016 ^18^. Para evaluar la precisión del método, se estimó la repetibilidad, para ello se realizó el análisis de réplicas (n = 10) del material de control de orina BIO-RAD bajo condiciones experimentales idénticas, se calculó la desviación estándar relativa (%DER). La veracidad del método se determinó comparando los resultados de cuatro muestras analizadas mediante el método VRAO con los obtenidos a través del método de referencia, AAVF (absorción atómica de vapor frío). Para comparar estadísticamente la diferencia entre ambos métodos, se realizó una prueba t pareada, previa verificación de la normalidad de los datos. Se consideró un nivel de significancia de p < 0,05.

Para la evaluación de la linealidad se construyó una curva de concentración de mercurio (II) versus respuesta con cinco puntos y tres réplicas por cada una de las concentraciones (2,5, 5, 10, 15 y 20 µg/L) en solución acuosa de HCl (c= 0,0125 mol L^-1^). Los LDD y LDC se analizaron con siete muestras de orina enriquecidas con mercurio (II) a una concentración de 2,5 µg L^-1^, se aplicaron las ecuaciones 1 y 2 respectivamente.

LDD = X + 3DS (Ecuación 1)

LDC = X + 10DS (Ecuación 2)

## HALLAZGOS

### Parámetros de optimización electroquímica

En la figura 2 se presentan los resultados de la optimización de los parámetros del sistema VRAO. El potencial óptimo para la deposición de mercurio (II) se observa en la figura 2A, cuyo pico de intensidad de corriente se registra a un potencial de 0 voltios (V), indicando la señal óptima para este parámetro. El tiempo de deposición muestra una relación lineal positiva con la intensidad de corriente (figura 2B); sin embargo, tiempos excesivamente prolongados pueden saturar el ET con mercurio, dificultando su desorción durante la etapa de oxidación. Por ello, se seleccionó un tiempo de deposición intermedio de 120 s.

**Figura 2:** Optimización de los parámetros de funcionamiento de la VRAO: (A) potencial de deposición, (B) tiempo de deposición, (C) potencial de paso, (D) amplitud de modulación y (E) frecuencia, en las respuestas voltamperométricas (corriente pico de desprendimiento) para mercurio (II) en una solución de 0,0125 mol L⁻¹ HCl. V: voltios.

El potencial de paso (figura 2C) influye en la velocidad de barrido del mercurio y mejora significativamente la sensibilidad de la VRAO, se seleccionó un valor óptimo de 0,008 V, ya que proporcionó la mejor respuesta analítica para la detección de mercurio (II). La amplitud afecta directamente la altura y el ancho de los picos de intensidad eléctrica; como se muestra en la figura 2D, valores superiores a 0,03 V en la amplitud de modulación generaron picos más cortos para el mercurio (II). En cuanto a la frecuencia (figura 2E), los ensayos indicaron que su incremento no afectó significativamente la intensidad de la corriente pico hasta los 150 Hz; sin embargo, a 200 Hz se registró una disminución notable. Por ello, se optó por una frecuencia de 150 Hz.

En la figura 3 se muestra la respuesta del sistema VRAO en intensidad de corriente (I_p_) con los parámetros óptimos ajustados. La I_p_ aumenta linealmente con el incremento de la concentración de mercurio (II) en un rango de 2,5 a 20 µg L^-1^ de mercurio (II) (n = 5) con un coeficiente de correlación (R^2^) de 0,992 y una ecuación de curva de calibración: I_p_ (µ A) = 0,8 C + 0,6. La desviación estándar de siete muestras de orina enriquecidas con mercurio (II) (c = 2,5 µg L^-1^) fue de 0,28. El LDD y LDC resultó 0,68 y 2,2 µg L^-1^, respectivamente.

**Figura 3:** Respuesta del sistema VRAO con parámetros optimizados: potencial de deposición de 0,0 V, tiempo de deposición de 120 s, amplitud de pulso de 0,03 V, potencial de paso de 0,008 V y frecuencia de 150 Hz. Curva de calibración para la determinación de mercurio (II) en un rango de concentraciones de 2,5 a 20 μg L⁻¹.

El resultado de repetibilidad para el método VRAO fue de 6,21 para %DER significativamente inferiores al umbral teórico de 21,38 calculado utilizando la ecuación de Horwitz (2/3 %DER Horwitz).

Los resultados obtenidos por VRAO y AAVF se resumen en la tabla 1. El valor de p obtenido fue 0,260, lo que indica que no hay diferencia significativa entre las concentraciones de mercurio entre ambos métodos.

**Tabla 1:** Determinación de mercurio en muestras de control de orina BIO-RAD (Nivel 1; Lote 69221) por los métodos VRAO y AAVF.

Muestra	VRAO (μg L-1)	AAVF (μg L-1)	Valor de p
1	27,0	28,2	
2	30,5	29,1	
3	29,1	31,7	
4	25,7	31,2	
Media (DE)	28,1 (2,1)	30,1 (1,7)	0,260^a^

a Prueba t de Student para muestras pareadas

VRAO: voltamperometría de redisolución anódica de onda cuadrada, AAVF: absorción atómica de vapor frío, DE: desviación estándar.

## DISCUSIÓN

Se evaluaron parámetros de desempeño de la VRAO, enfocados en la linealidad, precisión, veracidad, límites de detección y cuantificación para asegurar la confiabilidad del método. Los resultados obtenidos de las muestras de control de orina confirmaron el desempeño y la calidad del método en condiciones de laboratorio para la cuantificación de mercurio. El método analítico demostró una precisión satisfactoria, cumpliendo con los criterios establecidos por la Guía Eurachem (%DER_Horwitz < 45%) ^18^. De manera similar, los valores de precisión intermedia, expresados ​​como coeficiente de variación, resultaron menor al 5%, lo cual se puede considerar un valor aceptable para un método analítico cuantitativo.

En contraste, se ha informado valores DER (%) en condiciones de repetibilidad que oscilaron entre 0,3% y 3,6% utilizando un electrodo de microfibra de carbono modificado con nanopartículas de oro en muestras de agua ^19^. Sin embargo, se ha desarrollado un método para la digestión de mercurio en muestras de orina utilizando ácido concentrado a temperaturas elevadas; este método demostró una buena repetibilidad, como lo confirman las bajas desviaciones estándar (<11%). además, su análisis verificó que no se produjo pérdida de mercurio durante la digestión ácida ^20^, similar al método actual, lo que refuerza la aplicabilidad del enfoque propuesto para el análisis de mercurio en muestras de orina.

La repetibilidad observada del método de VRAO se puede atribuir a los parámetros electroquímicos, incluidos el potencial de deposición, el tiempo de deposición y la frecuencia ^21^. Estos parámetros se seleccionaron cuidadosamente para mejorar la acumulación de iones de mercurio en la superficie del electrodo, mejorando así la sensibilidad y la precisión de las mediciones. El uso de un electrodo de oro, conocido por sus propiedades electroquímicas estables, contribuyó aún más a la solidez del método ^22^. La concordancia de resultados entre VRAO y AAVF sugiere que el método voltamperométrico es capaz de lograr una precisión comparable a la técnica espectroscópica, a pesar de los desafíos inherentes asociados con las mediciones electroquímicas en matrices biológicas complejas.

Como limitaciones se debe reconocer que el presente estudio hace uso de controles comerciales de orina liofilizada, asimismo, no se ha determinado la vida útil del electrodo ni la deriva del sistema aplicado. La linealidad sólo contempla la respuesta en solución acuosa, no se ha estudiado el efecto de la matriz. Se necesitarán estudios futuros en muestras poblacionales para evaluar la aplicabilidad del método VRAO en una gama más amplia de condiciones y matrices para establecer su utilidad en diversos contextos analíticos.

En conclusión, este estudio desarrolló una metodología analítica aplicando la VRAO, se optimizaron los principales parámetros analíticos, y se realizó una validación preliminar para la medición de trazas de mercurio en agua y en orina. Los resultados demuestran que la VRAO es un método con una adecuada precisión, comparable al método de referencia AAVF y adecuados valores de LDM, LCM; sumado a la gran ventaja de ser un método portable, le otorga un alto potencial para su aplicación como herramienta en la evaluación de la exposición ocupacional al mercurio. Las investigaciones futuras deberían centrarse en optimizar los parámetros de la VRAO para detectar otros metales traza y evaluar su aplicabilidad en diversas muestras biológicas y ambientales. Además, la integración de la VRAO con tecnologías emergentes, como sistemas microfluídicos o sensores basados en nanomateriales, podría mejorar su sensibilidad y selectividad, allanando el camino para soluciones innovadoras en química analítica.
